# Rapid Generation of Barley Homozygous Transgenic Lines Based on Microspore Culture: *HvPR1* Overexpression as an Example

**DOI:** 10.3390/ijms24054945

**Published:** 2023-03-03

**Authors:** Zhiwei Chen, Qi Jiang, Guimei Guo, Qiufang Shen, Jun Yang, Ertao Wang, Guoping Zhang, Ruiju Lu, Chenghong Liu

**Affiliations:** 1 Shanghai Key Laboratory of Agricultural Genetics and Breeding, Biotechnology Research Institute, Shanghai Academy of Agricultural Sciences, Shanghai 201106, China; 2 Institute of Crop Science, College of Agriculture and Biotechnology, Zhejiang University, Hangzhou 310058, China; 3 National Key Laboratory of Plant Molecular Genetics, CAS Center for Excellence in Molecular Plant Sciences, Institute of Plant Physiology and Ecology, SIBS, Chinese Academy of Sciences, Shanghai 200032, China

**Keywords:** *Hordeum vulgare* L., microspore culture, transformation, doubled haploids, pathogenesis-related-1 gene

## Abstract

Obtaining homozygous lines from transgenic plants is an important step for phenotypic evaluations, but the selection of homozygous plants is time-consuming and laborious. The process would be significantly shortened if anther or microspore culture could be completed in one generation. In this study, we obtained 24 homozygous doubled haploid (DH) transgenic plants entirely by microspore culture from one T_0_ transgenic plant overexpressing the gene *HvPR1* (pathogenesis-related-1). Nine of the doubled haploids grew to maturity and produced seeds. qRCR (quantitative real-time PCR) validation showed that the HvPR1 gene was expressed differentially even among different DH_1_ plants (T_2_) from the same DH_0_ line (T_1_). Phenotyping analysis suggested that the overexpression of *HvPR1* inhibited nitrogen use efficiency (NUE) only under low nitrogen treatment. The established method of producing homozygous transgenic lines will enable the rapid evaluation of transgenic lines for gene function studies and trait evaluation. As an example, the *HvPR1* overexpression of DH lines also could be used for further analysis of NUE-related research in barley.

## 1. Introduction

Barley is the fourth largest cereal crop in the world and also one of the most important crops in China. It has been part of the modern agricultural industry technology systems of China since 2007 [1]. Cell engineering breeding based on anther and microspore culture is an important component of the barley breeding system and these techniques have been widely used in barley breeding and scientific research in China. This has been facilitated by improvements in microspore culture techniques, including genotype-independent microspore culture, and the combination of mutagenesis and hybridization with microspore culture to produce new DH lines [2,3,4,5].

Transgenesis is increasingly used in barley gene function analysis and breeding. However, it is time-consuming and laborious to obtain homozygous transgenic plants by traditional means. The production of doubled haploid (DH) plants from the T_0_ plants could substantially speed up the process, and transgenic DH plants could be selected without further consideration of heterozygosity. Thus, the production of DH plants would greatly improve the efficiency of transgenic-related research or breeding in barley and other crops.

The Green Revolution based on the incorporation of dwarfing genes into breeding programs and the use of inorganic chemical fertilization (especially nitrogen fertilizer) has led to huge increases in crop yields [6]. However, this mode of crop production is now facing many problems [7,8] and new strategies for improving crop yield while reducing N inputs are receiving more attention. Previously, we found a special response of the barley pathogenesis-related 1 (*HvPR1*) gene to low nitrogen stress in the barley cultivar BI-04, based on RNA-seq data (NCBI accession number: PRJNA400519) [9]. In this study, we aimed to combine transgenesis with microspore culture to generate doubled haploid (DH, homozygous) transgenic barley plants overexpressing the *HvPR1* gene, and verify its function in low nitrogen stress as well as its effects on nitrogen use efficiency (NUE). The workflow that was established will be beneficial to the study of gene function and breeding in barley, and also provide an example for other crops.

## 2. Results

### 2.1. Plant Transformation and Microspore Culture

**Figure 1:**
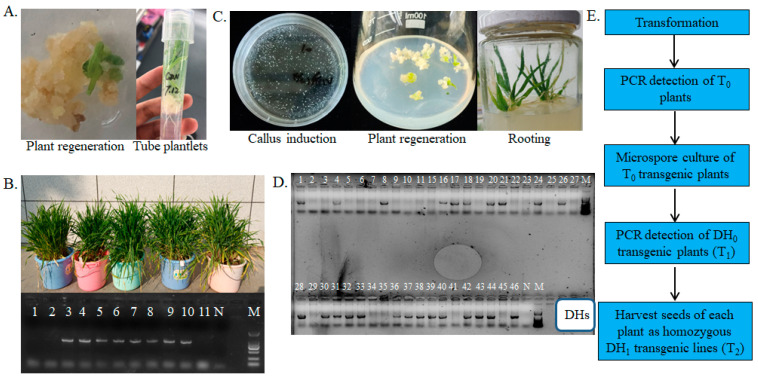
Schemes follow the same formatting. The workflow for the generation of homozygous transgenic barley plants by microspore culture. (**A**) Transformation in barley. (**B**) Identification of transgenic barley plants in the T_0_ generation by normal PCR; different lanes of the gel represent different T_0_ plants (see Supplementary Materials Figure S1). (**C**) Microspore culture of transgenic T_0_ barley plants. (**D**) Identification of transgenic barley doubled haploid (DH_0_) plants (T_1_); different numbers represent different DH_0_ plants from the 10th T_0_ plant (see Supplementary Materials Figure S2). (**E**) Summary of the workflow. “N” means the negative control sample (H_2_O); “M” means the marker of DNA ladders.

The HvPR1 gene was cloned into the modified binary plasmid pCAMBIA1301-FLAG with a hygromycin resistance marker gene (Table 1). The recombinant plasmid was transformed into Agrobacterium tumefaciens and the Agrobacterium tumefaciens with the recombinant plasmid was used for the infection of immature embryos of the barley cultivar Golden Promise for genetic transformation. In total, eighteen T_0_ plantlets were regenerated and transplanted in an artificial climate room, and eleven of these survived to form seedlings (Figure 1A,B). These seedlings were screened by PCR amplification of the hygromycin resistance marker gene, and eight of them were positive (Figure 1B). A total of 46 DH_0_ plants (T_1_) were obtained just from the 10th T_0_ transgenic plant, and 43 of them survived to form seedlings (Table 2; Figure 1C). Among these surviving seedlings, 24 were shown to be transgenic (Figure 1D), and 9 of these grew to maturity and produced seeds. Therefore, a method was established for the efficient and rapid generation of homozygous transgenic barley plants based on microspore culture (Figure 1E).

### 2.2. PCR Detection and qPCR Validation of Transgenic Plants

**Figure 2:**
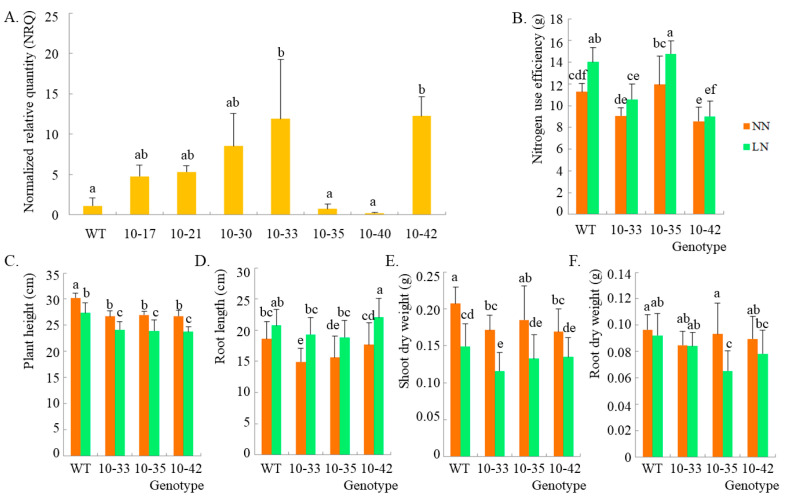
Gene transcription analysis and phenotypic investigation of transgenic seedlings. (**A**) Validation of HvPR1 transcription by qPCR. (**B**) Nitrogen use efficiency (NUE, NUE = SDW/SNC). (**C**) Plant height (PH). (**D**) Root length (RL). (**E**) Shoot dry weight (SDW). (**F**) Root dry weight (RDW). Means and standard deviations (standard errors for NRQ) are shown, and different letters indicate significant differences (*p* < 0.05, analyzed using the LSD test for all values). *n* = 3 for NRQ and NUE, respectively; *n* = 10 for PH, RL, SDW and RDW, respectively. WT: wild type; 10–33 and 10–42: the positive transgenic DH1 lines; 10–35: the negative transgenic DH_1_ line; NN: normal nitrogen supply (CK); LN: low nitrogen stress.

Considering the relatively small number of transgenic seedlings (T_0_ plants) and the statistics, PCR detection alone was used for transgenic detection. The transgenic rate was very high, indicating that the transgenic screening system was very effective. PCR and qPCR analysis were used together for the detection of transgenic DH_0_ lines. From the PCR detection, more than half of the plants were positive. However, fewer plants grew to maturity and produced seeds, indicating that some might not have completed chromosome doubling. This might have been caused by the barley genotype or weak growth due to the transgenesis. Seven transgenic DH_0_ lines with enough seeds (DH_1_ generation) were used for the qPCR analysis of the HvPR1 gene at seedling stage, and only two of them had significantly higher gene expression than the wild type (WT) (Figure 2A). This might be related to the microspore culture process and the differences between individual transgenic plants. Thus, the transgenic DH_1_ lines (T_2_) of 10–33 and 10–42 were taken for functional analysis of the HvPR1 gene.

### 2.3. Phenotypic Identification by Using HvPR1 Overexpression Plants under Low-Nitrogen Treatment

To verify the function of the HvPR1 gene under low nitrogen stress, the nitrogen use efficiency (NUE) and morphological traits were investigated under normal nitrogen (NN, control) and low nitrogen (LN) treatments at seedling stage (Figure 2). It was found that the NUE of WT and all DH_1_ lines increased under the low nitrogen treatment compared with the normal nitrogen treatment, but only WT and 10–35 (the negative transgenic DH_1_ line) were significant (Figure 2B). Comparing to the WT, it was found that the NUE of transgenic DH_1_ line of 10–42 was significantly lower than that of the WT under NN treatment, but the NUE of both 10–33 and 10–42 was significantly lower than that of WT under LN treatment (Figure 2B). In addition, there were no significant differences in NUE between WT and 10–35 under NN or LN treatment. For other traits, there were significant reductions in plant height (PH) and shoot dry weight (SDW) under the LN treatment compared with NN treatment in WT and all DH1 lines, and there were reductions in root dry weight (RDW) under the LN treatment compared to the NN treatment in WT and all DH_1_ lines, but this was only significant in 10–35. However, the root length (RL) was different, being significantly increased under LN treatment compared with NN treatment in all DH lines except the WT (Figure 2C–E). Compared with the WT, it was found that the PH of all DH_1_ lines was significantly reduced under both nitrogen treatments, while there were significant reductions in SDW in the 10–33 and 10–42 lines under NN treatment and significant reductions in SDW only in the 10–33 line under LN treatment. There were significant reductions in RDW only in the 10–35 line, and there were significant increases in RL in the 10–33 and 10–35 lines under NN treatment (Figure 2C–E). These results indicate that the inhibition of plant growth by LN treatment might be mainly reflected in shoots at first. The overexpression of the HvPR1 gene could inhibit plant growth even under the NN treatment, while the inhibition of NUE was mainly reflected under the LN treatment.

## 3. Discussion

The homozygosity of transgenes is necessary for phenotyping and trait evaluation. For a long time, scientists have tried to create homozygous transgenes quickly by genetically modifying haploid cells and then doubling them. Transformations based on anthers or microspores are most commonly used in barley [10,11,12,13]. However, the rapid generation of homozygous, transgenic T_0_ plants by the microspore culture of T_0_ pollens seemed to be the simpler option. Recently, the rapid generation of homozygous transgenic barley DH plants by anther culture of T_0_ pollen has also been reported [14]. However, microspore culture is more efficient and advantageous in producing DH plants [15]. Moreover, a microspore culture technology system that is genotype-independent and has a high rate of regeneration of green plants is now established, enabling more than one thousand DH plants to be derived from a single parent plant. This technology has become the main technical approach for barley DH line production used in scientific research and breeding in China [16]. Therefore, it may be more suitable to develop homozygous barley transgenics based on the microspore culture of T_0_ pollen.

The *Agrobacterium*-mediated transformation of immature embryos of the variety Golden Promise is very successful and widely used [13]. In our study, eight of eleven T_0_ plants were positive by PCR detection (with a rate of about 73%), showing that the *Agrobacterium*-mediated transformation was very efficient. However, we only obtained DH plants from T_0_-6 and T_0_-10 plants. This was most likely due to the poor state of growth of these transgenic plants. Moreover, many transgenic DH_0_ lines from the T_0_-10 plants were sterile (with a rate of 62.5%), as were non-transgenic DH_0_ lines. The reduced fertility might be due to transformation [10]. Additionally, high rates of haploids were also observed in anther culture [14]. Normally, after transplanting in the nursery, regenerated seedlings are treated with 0.1% colchicine for chromosomal doubling, but this step is currently omitted due to the high rate of spontaneous chromosomal doubling of barley and the high efficiency of our culture system [3,15]. In this case, the artificial chromosomal doubling was necessary to improve the fertility of the regenerated green plants from the microspore culture of T_0_ pollen. In addition, there were high rates of albino plants in the anther culture [13,14], while this was not a problem in microspore culture. Considering the high efficiency of microspore culture and the difficulty of transformation of microspores, the establishment of a homozygous transgenic system based on the microspore culture of T_0_ pollen, especially genotype-independent microspore culture for the rapid generation of homozygous transgenic barley plants.

Research on the *PR1* gene mainly focuses on disease resistance in plants, while recent studies have shown that it may be involved in a variety of abiotic and biotic stresses [17,18]. However, the role of the *PR1* gene in low nitrogen tolerance or nitrogen use efficiency in barley has not been reported. In this study, we found that the *HvPR1* gene inhibited nitrogen use efficiency under low nitrogen treatment, and overexpression of *HvPR1* also affected barley growth even under a normal nitrogen supply. Therefore, the acquisition of homozygous transgenic barley lines will be of great benefit for further research. At the same time, we also found that only two DH_1_ lines showed significant upregulation of the *HvPR1* gene. This suggested the possibility of gene silencing or transgene loss, which had been reported previously [19]. This was not observed in anther culture using normal RT-PCR validation [14].

## 4. Materials and Methods

### 4.1. Gene Cloning and Overexpression Vector Construction for the HvPR1 Gene

Leaves of barley cultivar BI-04 were used for RNA extraction. RNA extraction and cDNA synthesis were performed according to Chen et al. [20]. The cDNA was used as the template to clone the full length of *HvPR1* cDNA (GenBank: Z21494.1). Its coding region, minus the stop codon, was further sub-cloned into the modified binary plasmid pCAMBIA1301-FLAG using the LR cloning system (Thermo Fisher Scientific, Waltham, MA, USA), according to He et al. [21]. pCAMBIA1301-FLAG also contains a hygromycin resistance marker gene. The primers used for gene cloning and vector construction are listed in Table 1.

### 4.2. Barley Transformation of HvPR1 Gene Mediated with Agrobacterium

The recombinant plasmid was transformed into the *Agrobacterium tumefaciens* strain AGL1 and *Agrobacterium tumefaciens* with the recombinant plasmid used for the infection of immature embryos of the barley cultivar Golden Promise for genetic transformation according to Shen et al. [22]. The single colony of *Agrobacterium* AGL1 within the *HvPR1* vector was cultured in liquid MG/L medium within Rifampicin and Kanamycin at 28 °C until the OD_600_ value was around 1.5. Barley spikes of Golden Promise were collected at 15 DAF, when the embryos were approximately 1–2 mm in diameter. Then, the immature seeds were sterilized with 70% ethanol and 10% sodium hypochlorite, respectively. The embryos were isolated from these sterilized seeds, and transferred into CI medium any antibiotics (the scutellum was placed upside). Then, a droplet of the prepared *Agrobacterium* was inoculated to each scutellum, then transferred to new plates within CI medium for cocultivation (the scutellum was placed downside). Approximately 3 days later, these co-cultured embryos were transferred to new CI medium with the antibiotics Timentin and Hygromycin, as well as copper for callus induction and selection. Yellowish, loosely structured, and distinctly granular calli were selected and transferred to T medium for two weeks. Then, calli with green regions or developing shoots were selected and transferred to modified B1 medium for regeneration. The regenerated barley T_0_ plants from transformation were transplanted in an artificial climate room, and these seedlings were confirmed by PCR amplification of the hygromycin resistance marker gene. This pair of primers for the hygromycin resistance marker gene is also listed in Table 1.

### 4.3. Microspore Culture of Transgenic Barley Plants

The confirmed transgenic barley plants (T_0_) were further used as donor plants for microspore culture according to Lu et al. (Table 2) [3]. Spikes of the transgenic barley plants were collected at mid- to late-uninucleate microspore stage and stored in a refrigerator at 4 °C for 2–3 weeks. These spikes were surface-sterilized with 10% NaClO for 10 min and then rinsed with sterilized water. Approximately 300 anthers were separated and placed into 50 mL centrifuge tubes, and then the isolation buffer was added for microspore isolation. Microspore isolation buffer contained 60 g/L mannitol, 1.1 g/L CaCl_2_, 0.976 g/L MES and 20 mg/L colchicine. The isolated microspores were suspended with the induction medium and adjusted to a density of 5.0 × 10^5^ microspores/mL. The induction media were mainly based on N6 medium with some modifications. A total of 1 mL or 3 mL of this microspore mixture was transferred into a Petri dish with size of 35 mm × 12 mm (smaller Petri dish) or 60 mm × 15 mm (bigger Petri dish) in size, respectively, then the Petri dishes were sealed with parafilm and incubated in darkness at 25 °C for callus induction. After approximately 19 days, the calli were transferred into 100 mL triangle flasks with 50 mL differentiation medium for plant regeneration at 25 °C under fluorescent lights with a 16 h photoperiod. The differentiation medium was 2/3 MS medium supplemented with kinetin at 1.5 mg/L, 6-BA at 0.5 mg/L, and NAA at 0.05 mg/L, with 30 g/L maltose as the carbon source, and solidified with agar at 6 g/L. Approximately two weeks later, the regenerated shoots were then transferred into 100 mL triangle flasks with 50 mL rooting medium at 25 °C under the fluorescent lights with a 16 h photoperiod for approximately one month. The rooting medium was 1/2 MS medium supplemented with MET (4 mg/L) and NAA (0.05 mg/L), with 30 g/L sucrose as the carbon source and solidified with agar at 6 g/L. Then, the regenerated plantlets (or R_0_ plants) were cultured in the 1/2 Hoagland nutritional liquid for 2–3 weeks in an artificial climate room. The microspore isolation buffer and introductive mediums were sterilized by filter sterilization, while the regeneration and rooting mediums were sterilized by high-temperature autoclaving (0.11 Mpa, 121 °C for 15 min), and all mediums were adjusted to pH 5.8. The seedlings after transplanting in the nursery were cultivated in pots within soil for harvesting seeds in artificial climate room. These plants were different transgenic DH_0_ lines and were analyzed by the PCR amplification of the hygromycin resistance marker gene as mentioned above.

### 4.4. Gene Expression Analysis of HvPR1 by qPCR

The WT and transgenic DH_0_ lines that could grow to maturity and produce seeds (DH_1_) were used for qPCR analysis. RNA extraction, cDNA synthesis, and qPCR procedures were mainly conducted according to Chen et al. [20]. Primers used for the *HvPR1* gene and reference genes are listed in Table 1. The primers for the *HvPR1* gene were designed by Primer-BLAST using the NCBI website, while primers for reference genes were directly taken from Chen et al. [9]. The two most stable reference genes, as calculated by geNorm, were used for normalization: *HvGAPDH* (glyceraldehyde-3-phosphate dehydrogenase) and *HvTUBB6* (beta tubulin 6). The NRQ (normalized relative quantity) was calculated using this formula (NRQ = $E_{\mathrm{HvPR}1}^{-\mathrm{Cq},\;\mathrm{HvPR}1}/\sqrt{E_{\mathrm{HvGAPDH}}^{-\mathrm{Cq},\mathrm{HvGAPDH}}\cdot E_{\mathrm{HvTUBB}6}^{-\mathrm{Cq},\mathrm{HvTUBB}6}}$). The transformed Cq′ values (Cq′ = log2 (1/NRQ)) were used for the comparisons, and the significant differences in gene expression between the wild type (WT) and the transgenic DH_1_ shoots were evaluated by the LSD test at the 0.05 level (*p* < 0.05). There were three biological replicates for WT and each transgenic DH_1_ line.

### 4.5. Plant Growth and Low-Nitrogen Treatment

Wild-type (WT) barley seeds and the DH_1_ seeds (10–33, 10–35 and 10–42) were sterilized with 1% NaClO for 30 min, then rinsed with water. After soaking in water for 6 h, seeds were germinated at 25 °C for one week. Seedlings were cultured in an artificial climate chamber, and the growth conditions and nitrogen treatments were mainly conducted according to Chen et al. [23]. NH_4_NO_3_ was used as the nitrogen source, and there were two nitrogen treatments: the normal nitrogen (NN) supply with 1.43 mM NH_4_NO_3_ (control) and low nitrogen (LN) stress with 0.24 mM NH_4_NO_3_. The low nitrogen treatment was started from the 3- to 4-leaf stage of seedling development for two weeks. The plants were then harvested, and plant height (PH) and root length (RL) were measured directly. Then, shoots (above grounds) and roots were separated and collected, respectively. There were ten biological replicates for the WT and DH_1_ lines, respectively.

### 4.6. Biomass and Total Nitrogen Measurement

The separate shoots and roots were incubated at 105 °C for 1 h and dried at 80 °C for 2 days until constant weight; then, they were weighed for shoot dry weight (SDW) and root dry weight (RDW), respectively, using an electronic analytical balance. Every three dried shoots (three biological replicates) formed a new biological replicate, and nine dried shoots formed a total of three new biological replicates. The dried shoots were ground to powder and approximately 0.2 g of the dry powder of each sample was digested (H_2_SO_4_-H_2_O_2_) for total nitrogen determination using the Kjeldahl method. Based on the different definitions, the NUE was evaluated by the shoot biomass [24,25]. The shoot nitrogen content (SNC) (%, g N/100 g SDW) of each sample could be directly calculated from the total nitrogen detection. The NUE was calculated as follows: NUE (g) = SDW/SNC. This indicated that the higher dry weight and lower nitrogen content would result in higher N use efficiency.

## 5. Conclusions

In summary, we established a method for the efficient and rapid generation of homozygous transgenic barley plants based on microspore culture. This method requires only one generation to obtain homozygous transgenic plants, and although microspore culture takes some time, far more time is saved because of the additional generations required for traditional methods. The planting scale is also small, which can use space more efficiently and save labor. Meanwhile, we also obtained homozygous *HvPR1*-overexpressing plants and demonstrated the role of this gene in response to low nitrogen stress in barley.

## Figures and Tables

**Table 1:** Primers used in this study.

Description	Primer Name	Sequence (5′-3′)	Origin
*HvPR1* cloning and overexpression vector construction	HvPR1-F	TTATACACCGAACCGAGAATG	This study
	HvPR1-R	GCATCACGGTTAGTATGGTTT	
	HvPR1-BamH I-F	CGGGATCCATGCAGACGCCCAAGCTAG	
	HvPR1-EcoR I-R	GGAATTCTTAGTATGGTTTCTGTCCAATGATA	
	HvPR1-FLAG-EcoR I-R	GGAATTCGTATGGTTTCTGTCCAATGATATTC	
Transgenic detection	Hyg-F	TGAAAAAGCCTGAACTCACCG	Ertao Wang’s lab
	Hyg-R	TATTTCTTTGCCCTCGGACG	
qPCR	HvPR1-q-F	ATCAACGACTGCAAGCTCCA	This study
	HvPR1-q-R	GTCCTTCTTCTCGCTCACCC	
	HvTUBB6-F	TCCCGAACAATGTCAAGTCA	[9]
	HvTUBB6-R	GTGGAGTTGCCAATGAAGGT	
	HvGAPDH-F	AAGCATGAAGATACAGGGAGTGTG	
	HvGAPDH-R	AAATTTATTCTCGGAAGAGGTTGTACA	

**Table 2:** The summary of the microspore culture of transgenic plants.

Code of Transgenic T_0_ Lines	Number of Spikes for Anther Separation	Tubes of Separated Anthers	Petri Dishes within Microspore Mixtures	Petri Dishes with Inductive Calli	Number of Regenerated Plants
T_0_-3	44	4	Contaminated during callus induction		
T_0_-4	41	5	3 smaller Petri dishes	No calli	
T_0_-5	29	3	1 bigger Petri dish and 2 smaller Petri dishes	Less than 15 mg and 5 mg callus per dish of the bigger Petri dish and the 2 smaller Petri dishes, respectively	No plants
T_0_-6	47	5	5 bigger Petri dishes	Less than 15 mg callus per dish of the 5 bigger Petri dishes	1 plant
T_0_-7	15	2	Contaminated during callus induction		
T_0_-8	42	4	1 bigger Petri dish and 3 smaller Petri dishes	Less than 15 mg and 5 mg callus per dish of the bigger Petri dish and the 3 smaller Petri dish, respectively	No plants
T_0_-9	25	3	1 bigger Petri dish	Less than 15 mg callus per dish of the bigger Petri dish	Contaminated during differentiation
T_0_-10	24	4	4 bigger Petri dishes	Approximately 90 mg callus per dish of 3 of the 4 bigger Petri dishes	46 plants

Note: Any contamination was discarded and no plants were obtained.

## Data Availability

https://www.ncbi.nlm.nih.gov/bioproject/?term=PRJNA400519 (accessed on 28 September 2018).

## Supplementary Materials

The following supporting information can be downloaded at: https://www.mdpi.com/article/10.3390/ijms24054945/s1.
