# Supplementary material for: Rapid Generation of Barley Homozygous Transgenic Lines Based on Microspore Culture: HvPR1 Overexpression as an Example
Source: Int J Mol Sci. 2023 Mar 3;24(5):4945. doi: 10.3390/ijms24054945 (PMC10003194; doi:10.3390/ijms24054945)
Supplement: Supplementary file 1 [file ijms-24-04945-s001.zip › ijms-2122155-supplementary.pdf]

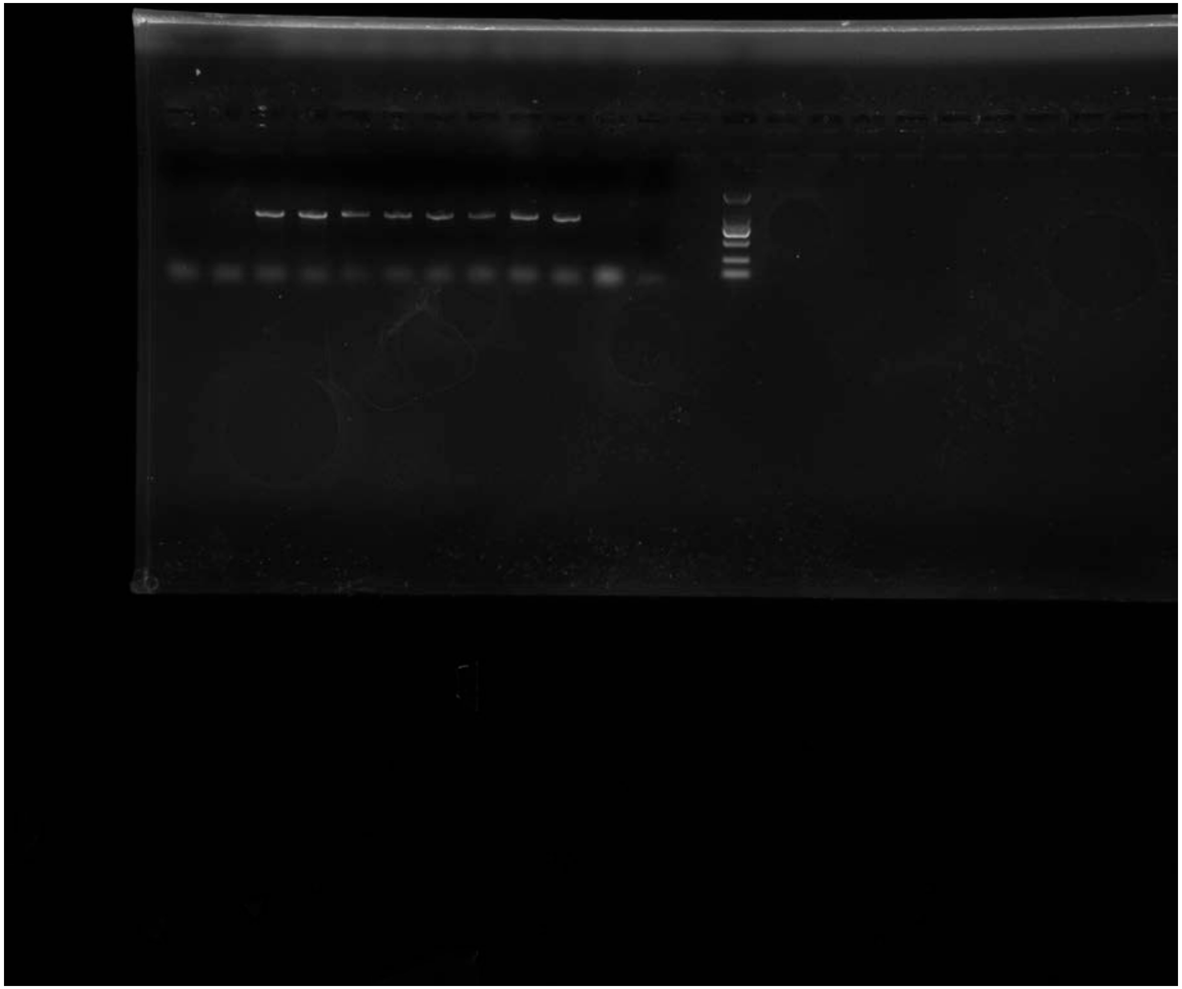

Figure S1. Gel picture of PCR products for detection of transgenic T_0 plants (The original picture for Figure 1B)

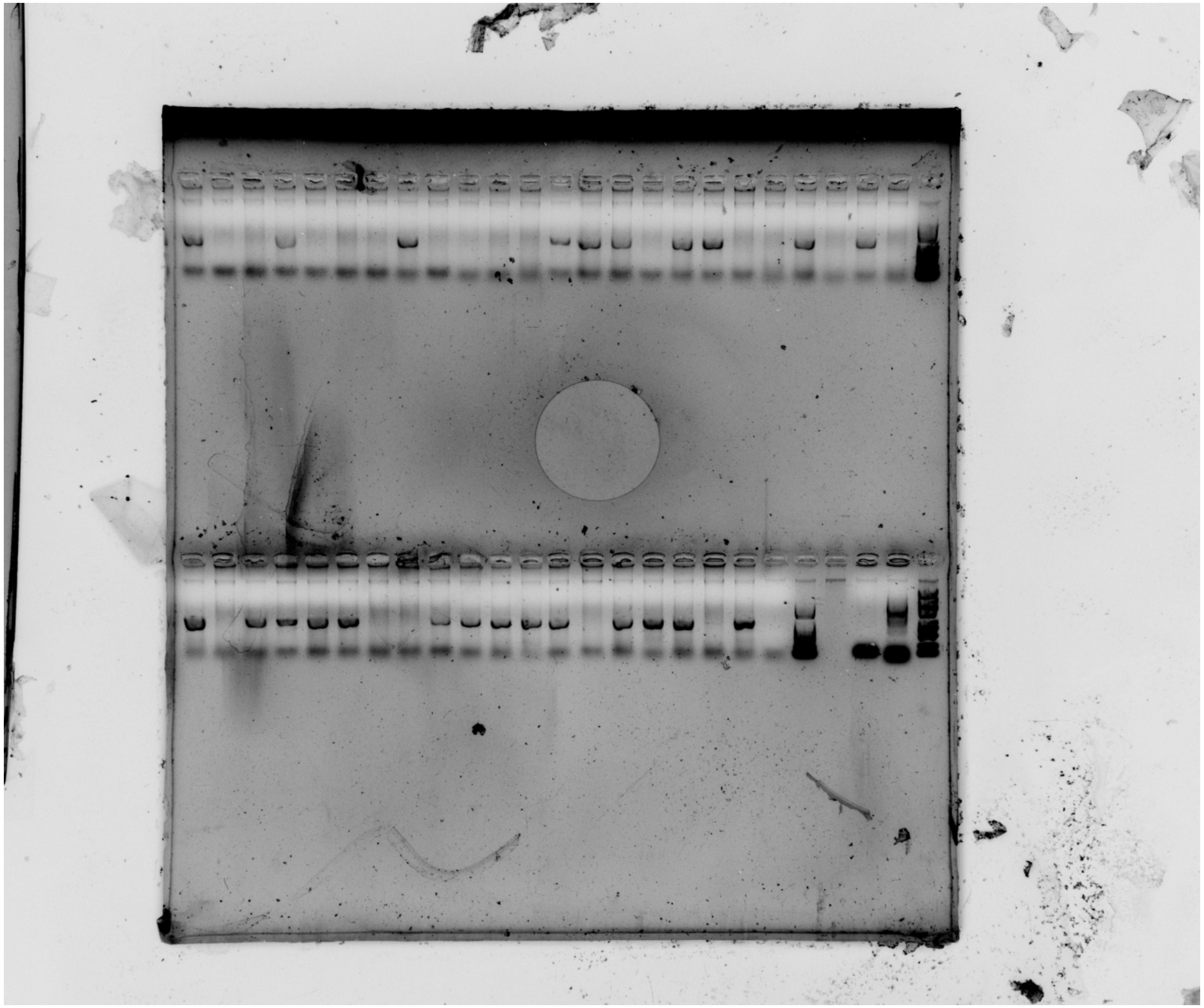

Figure S2. Gel picture of PCR products for detection of transgenic DH_0 plants. (The original picture for Figure 1D, and the products of last three lanes are not relevant to this experiment.)
